# Impact of Short‐Term High Temperature on *Microplitis manilae* (Hymenoptera: Braconidae) Survival, Fecundity and Progeny Development

**DOI:** 10.1002/ece3.71626

**Published:** 2025-06-18

**Authors:** Peng Ren, Xue‐Yuan Di, Bin Yan, Xiu‐Xian Shen, Shuang Tian, Mao‐Fa Yang, Hui‐Zi Wu

**Affiliations:** ^1^ Guizhou Provincial Key Laboratory for Agricultural Pest Management of the Mountainous Region, Institute of Entomology Guizhou University Guiyang Guizhou China; ^2^ College of Tobacco Science Guizhou University Guiyang Guizhou China; ^3^ Zunyi Branch Company of Guizhou Tobacco Company Zunyi Guizhou China

**Keywords:** biological control, global warming, high‐temperature stress, *microplastic manilae*, *Spodoptera litura*

## Abstract

With global warming and an increase in the frequency of extreme heat events, uncertainty has arisen about the survival and fertility of insects that are enemies in the wild. In this study, we employed the generalized linear model (GLMs) for analysis to elucidate the effects of short‐term high temperatures on the survival, fecundity, and progeny of *Microplitis manilae* Ashmead (Hymenoptera: Braconidae), a key parasitic natural enemy of early instar larvae of *Spodoptera litura* Fabricius (Lepidoptera: Noctuidae). We conducted laboratory tests to assess the survival and lifespan of *M. manilae* adults at high temperatures of 35°C, 37°C, and 39°C, with exposure durations of 1, 3, 5, 7, and 9 h. We also evaluated the fecundity and longevity of the F_1_ generation adults, as well as the development of the F_2_ generation, following exposure at the same high temperatures for 5 h. The results indicated that *M. manilae* adult stages were significantly affected by elevated temperatures across different exposure durations, particularly when the duration exceeded 5 h. Under different high‐temperature exposures for 5 h, fecundity and longevity were significantly suppressed in the F_1_ generation, along with impaired growth and development in the F_2_ generation when the temperature exceeded 39°C. This study reveals the effects of short‐term high‐temperature stress on the survival, reproduction, and progeny development of *M. manilae* . It provides a theoretical basis for understanding the biological evolution mechanism of *M. manilae* in response to thermal stress and the biological control of pests by *M. manilae* in high‐temperature seasons.

## Graphical abstract

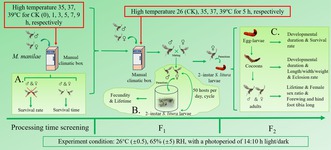

## 1 Introduction

Global warming is rising; in the last century, global temperatures increased by about 0.68°C. Furthermore, global average temperatures are projected to rise by 1.5°C–4.5°C by the end of this century (Easterling et al. 2000; Walther et al. 2002). The increase in global climate temperatures coincides with an increase in the incidence of extreme heat events (Kiritani 2013). As poikilothermic organisms, insects are particularly susceptible to disruptions in their survival, development, and fecundity due to external heat environmental conditions (Pecl et al. 2017). Short‐term exposure to high temperatures significantly reduces the survival and fecundity of the adult white butterfly pupal parasitoid wasp *Pteromalus puparum* (Xiong et al. 2024). Moreover, exposure to 36°C, 38°C, and 40°C for 2, 3, and 6 h negatively affected the growth, development, and parasitism of the Japanese *coccophagus* wasp *Coccophagus japonicus* (Sun et al. 2024). Short‐term high temperatures affect the parents of parasitic wasps and the development of their offspring. Following exposure to high temperatures for a short term, the development of the *mirus* encyrtid wasp *Ooencyrtus mirus* progeny is affected (Nancy et al. 2022), while the survival rate of the Red‐bellied parasitic wasp *Microplitis rufiventris* progeny is significantly reduced (Elsabrout et al. 2021). Additionally, at high temperatures, the pupal weights of the progeny of the *Dendrolimus* punctatus' *trichogramma Trichogramma euproctidi* differ significantly (Tabebordbar et al. 2022), whereas the wing shape of the progeny of the fruit fly *Drosophila melanogaster* appears curled and spherical (Williams et al. 2003). High‐temperature stress also affects the sex ratio of offspring. After being exposed to a high temperature of 43°C–45°C for 1 h, the silverleaf whitefly *Bemisia tabaci* showed a decline in the offspring emergence rate and a decrease in the female sex ratio (Cui et al. 2008). Therefore, studying the effects of high temperature on insects, including parasitic wasps, can help us understand the adaptive responses of insects to thermal stress and how they affect life history traits in their offspring. Focusing on parasitoids specifically will enable us to more effectively deploy natural enemies in pest control.

**FIGURE 1:**
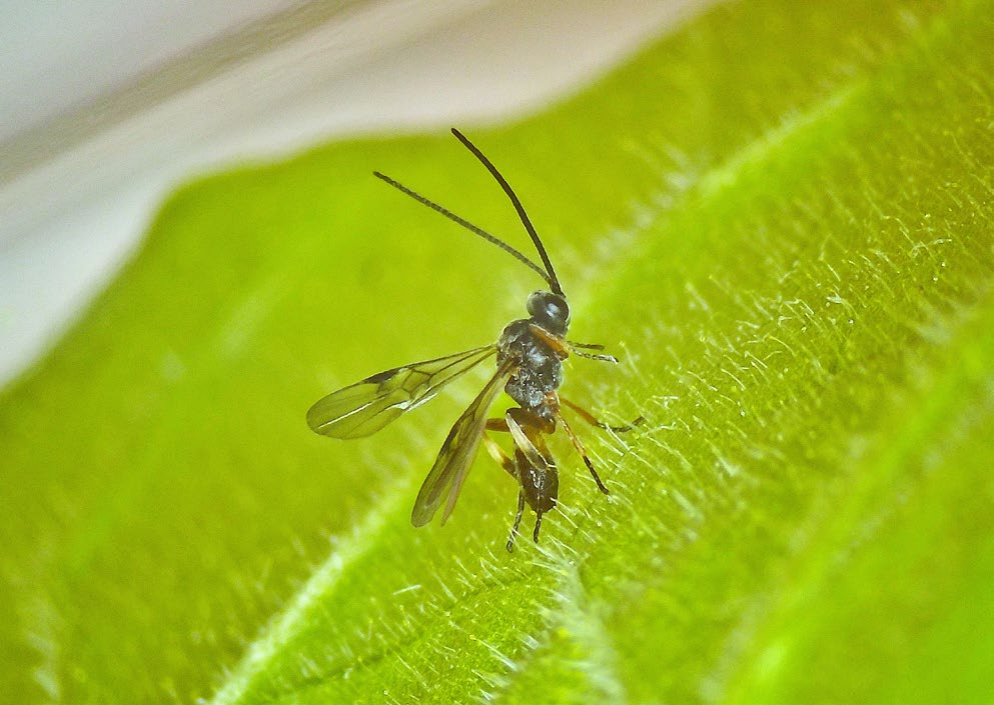
*M. manilae* (adult).

The braconid wasp *Microplitis manilae* Ashmead (Hymenoptera: Braconidae) is a dominant solitary endoparasitic parasitoid of the early instar larvae of pests of Noctuidae in Lepidoptera (Ando et al. 2006; Qiu et al. 2012). Figure 1 shows the morphology and typical state of adult *M. manilae* in its natural habitat. *M. manilae* is distributed mainly in tropical and subtropical regions, including southern China, Japan, Southeast Asia, and Indonesia (Ando et al. 2006; Ghafouri and Butcher 2023; Yan et al. 2023). It can parasitize important agricultural pests, such as several species of armyworm moths *Spodoptera litura* , *S. exigua* , and *S. frugiperda* (Ando et al. 2006; Qiu et al. 2013; Tian et al. 2024). This species can parasitize most of the first‐instar to third‐instar larvae of noctuid moths, with a particular preference for second‐instar larvae (Qiu et al. 2013). Additionally, among the various noctuid moth hosts, *M. manilae* preferentially parasitizes *S. litura* . For example, in Vietnam, *S. litura* larvae on soybean plants serve as their dominant hosts (Nakai and Cuc 2005), whereas in Japan, they also exhibit a preference for *S. litura* (Ando et al. 2006). Global warming has increased the frequency of extreme hot weather in tropical and subtropical regions (Freychet et al. 2021). Consequently, *M. manilae* populations are highly likely to experience thermal stress due to an increase in temperatures in their surrounding environment. Changes in ambient temperatures can significantly affect the development and fecundity of *M. manilae* (Qiu et al. 2012). The development time for egg, larvae, pupae, and the entire immature stage of *M. manilae* , as well as adult longevity, decreases, while survival and fecundity start declining when the ambient temperature increases from 17°C to 32°C (Fang et al. 2014). Additionally, lowering the temperature from 30°C to 20°C significantly extends the longevity of *M. manilae* adults (Xing et al. 2022). Thus, understanding the response of *M. manilae* to high‐temperature thermal stress can provide valuable insights for the use of natural enemies in pest control in summer.

Generalized linear models (GLMs) are flexible statistical methods widely used in biology to analyze population growth and the impacts of ecological factors on biological traits (Rathouz and Gao 2008). Researchers can employ GLMs to consider environmental factors as independent variables and use different models for different insect developmental parameters, such as longevity and fecundity, which are regarded as dependent variables, thus helping researchers understand the response relationships between insect biological characteristics and various factors in depth (Herrmann et al. 2023).

To investigate the effects of short‐term high temperatures on the survival and fecundity of *M. manilae* , we conducted laboratory tests and examined the survival and lifespan of adult *M. manilae* at 35°C, 37°C, and 39°C for 1, 3, 5, 7, and 9 h. Additionally, we evaluated the fecundity and longevity of the adults in the F_1_ generation and the growth and development of the F_2_ generation following exposure to the same high temperatures for 5 h. These findings provided a theoretical basis for more effectively using natural enemies in pest control.

## 2 Materials and Methods

### 2.1 Host and Parasite Feeding

*Spodoptera litura* was purchased from Yunke Biotechnology Company and reared in an artificial climate chamber (photoperiod: 14/10 h light/dark cycle; relative humidity: 65% ± 5%; temperature: 26°C ± 0.5°C) at the Guizhou Natural Enemy Propagation Center, Ministry of Agriculture and Rural Affairs, Guizhou University, using Chinese cabbage (variety: Guizhou Qianbai No. 9) plants as rearing plants. Two hundred egg masses of *S. litura* were placed in a round plastic box (13 cm high, 17.8 cm diameter) for feeding. Cabbage leaves were added to the box when the eggs hatched. We changed cabbage leaves regularly and cleaned up larval droppings. The petri dish (2.2 cm high, 10 cm diameter) was collected after pupation, and water was periodically sprayed to moisturize. After the pupae emerged, 5–10 pairs of male and female adults mated in round plastic boxes and fed on a 10% honey water solution. After the female adults had laid eggs, the eggs were transferred to a new round plastic box for rearing and breeding the next generation. *S. litura* was continuously bred in the laboratory for at least 30 generations.

Cocoons of *Microplitis manilae* were collected in August 2022 from asparagus plants in a field in Mochong Town, Duyun City, Qiannan Buyi, and Miao Autonomous Prefecture, Guizhou Province (107°22′52.3″ E, 25°56′03.7″ N) and were transported to the laboratory. The rearing conditions are the same as those for rearing *S. litura* , with lower‐instar *S. litura* larvae used as hosts. Two to three pairs of male and female adult *M. manilae* were allowed to mate in a test tube (9.5 cm high, 2.4 cm diameter) for 24 h. Then, they were transferred to a round plastic box (13 cm high, 17.8 cm diameter) with about 100 2nd‐instar *S. litura* larvae for parasitism. 10% honey water solution and fresh Chinese cabbage leaves were provided to rear both the parasites and the hosts. After being parasitized for 24 h, the male and female adult *M. manilae* were removed and the parasitized larvae of *S. litura* were reared in the plastic box until the cocoons of *M. manilae* formed. Then the cocoons were collected in plastic test tubes to be reared for the next generation and propagated similarly. *M. manilae* were continuously bred in the laboratory for at least 30 generations.

### 2.2 Experimental Temperature Gradient Design

**FIGURE 2:**
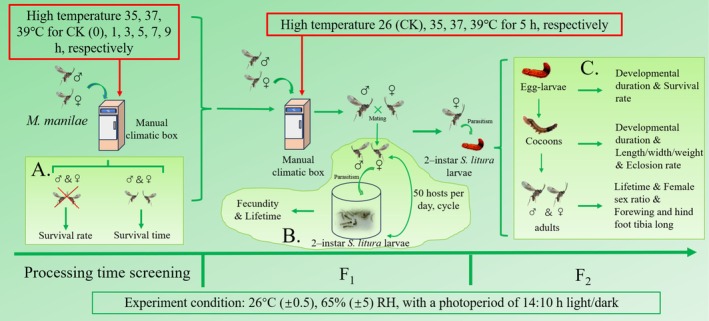
Experimental design flow chart. A, B, and C represent the three experiments in this paper respectively.

Since *M. manilae* lives in tropical and subtropical areas, the average extremely high temperature in summer is 35°C–39°C and sometimes even exceeds 40°C. The pre‐experimental results show that 39°C is the maximum temperature of adult *M. manilae* and that it cannot survive for more than 1 h at 40°C (See “Raw data” in Supporting Information). Therefore, the experimental temperature gradient was set at 35°C, 37°C, and 39°C. The treatment time was set to the common high‐temperature duration during the day. For the specific design, see the experimental technique (Figure 2).

### 2.3 Effects of High‐Temperature Exposure Time on the Survival of *M. manilae* Adults

In the laboratory (photoperiod: 14/10 h light/dark cycle; relative humidity: 65% ± 5%; temperature: 26°C ± 0.5°C), one‐day‐old unmated adult females and males of *M. manilae* were separated into two test tubes (9.5 cm high, 2.4 cm diameter), with 10 adults per tube (male or female). Then, they were placed in constant‐temperature manual climatic boxes (RXZ—380A, Ningbo Jiangnan Instrument Factory) set at temperatures of 35°C, 37°C, and 39°C for treatment durations of 1, 3, 5, 7, and 9 h, respectively. The humidity in the climate boxes was maintained at 65% ± 5%. Throughout the treatment phase, the photoperiod was set to the light phase. After the high‐temperature‐treated male and female adults were removed, the number of surviving individuals was recorded (after high‐temperature treatment, individuals who did not regain their activity within 1 h at room temperature 26°C ± 0.5°C were considered dead). The surviving female and male adults were reared normally until death in the laboratory, and the total survival time was recorded. A control (CK) experiment was performed without high‐temperature treatment. The parasitic wasps were provided with a 10% honey water solution throughout the experiment. Each experiment was repeated 5 times and carried out simultaneously.

### 2.4 Effects of High Temperature on the Fecundity and Longevity of F_1_ Generation Adults of *M. manilae*

One‐day‐old unmated F_1_ generation adult females and males of *M. manilae* were treated separately at 26°C (CK), 35°C, 37°C, and 39°C for 5 h. The conditions for high‐temperature treatment were the same as those used in previous experiments, and the high‐temperature exposure time was set according to previous experimental screening results. After the high‐temperature treatment, the plants were moved to the normal temperature of 26°C ± 0.5°C, the surviving male and female adults (in the same temperature treatment) were allowed to mate at a ratio of 1:1 for 24 h, and their fecundity was tested.

One pair of F_1_ generation *M. manilae* male and female adults (mated) treated at 26°C (CK), 35°C, 37°C and 39°C for 5 h was selected and placed in a round plastic box (13 cm high, 17.8 cm in diameter). Fifty second‐instar *S. litura* larvae were provided for regular parasitization daily. Appropriate cabbage leaves and 10% honey water solution were placed in the plastic box, and the parasitized hosts were replaced with new ones after 24 h of parasitism until the male and female adults died. The longevity of male and female adults was recorded. The caterpillars replaced after parasitization were fed normally every day, the larvae were observed every day until the parasitic wasps successfully formed cocoons, and the summary cocoon quantity of the parasitic wasps was recorded. Furthermore, the larvae that died during the experiment were dissected under a stereomicroscope (AO‐HK830‐0318, Shenzhen Oscilloscope Optical Instrument Co. Ltd.) to determine whether they were parasitized. Since newly parasitized larvae could not be distinguished, the total number of daily ovipositions was calculated by adding the number of cocoons and the number of deaths due to parasitism. Each experiment was replicated 10 times and carried out simultaneously.

### 2.5 Effects of High Temperature on F_2_ Generation Development in *M. manilae*

The F_1_ generation male and female adults were treated at 26°C (CK), 35°C, 37°C, and 39°C for 5 h (The high‐temperature treatment protocol matched that of the prior F_1_ experiment, aiming to assess transgenerational effects following high‐temperature exposure). The treated male and female adults (Same temperature treatment) were mated 1:1 for 24 h. One to two adult females that have mated are used to parasitize a single second‐instar *S. litura* larva in a round plastic box (13 cm high, 17.8 cm in diameter). When the female adult ovipositor was found to be inserted into the host body, it was judged to have parasitic success. The parasitized larvae were transferred to a plastic pudding box (height: 3.1 cm; diameter: 4.0 cm) for single‐head feeding. Each group treated 20 larvae, and the developmental time of the egg‐larval stage (both the egg and larval stages of endoparasitic wasps cannot be observed in the host stage and are collectively referred to as the egg‐larval stage), cocoon stage, and adult stage of F_2_ generation were observed and recorded. The egg‐larval survival, emergence rates, and female sex ratios were calculated, and cocoon weights were measured via an electronic balance (FA1004N, Shanghai Jinghai Instrument Co. Ltd.). The length and width of the cocoon and the length of the adult forewing and hind tibiae were measured via stereomicroscopy. Each experiment was replicated 5 times and carried out simultaneously.

### 2.6 Statistical Analysis

The indices recorded were as follows: the survival rate (%) = total number of individuals after treatment/total number of individuals before treatment ×100%; the longevity (days) = sum of the longevity of individuals/total number of individuals; the development time (days) = sum of the development time of individuals/total number of individuals; the emergence rate (%) = total number of emergence/total number of cocoons ×100%; and the female sex ratio (%) = total number of females emerged/total number of emergence ×100%.

All the data were analyzed via SPSS 27 (SPSS 27.0, IBM) software. The Kolmogorov–Smirnov test and Shapiro–Wilk test were used to test the normality and homogeneity of variance of all the data. The data under different high‐temperature treatments were analyzed via generalized linear models (GLMs), in which adult survival time, F_1_ generation adult's longevity, F_2_ generation cocoon length, width, and weight, and F_2_ generation adult's forewing and hind‐tibia length were analyzed via a GLM normal distribution model. Additionally, Cox Proportional Hazards models were used to further analyze the effects of different high‐temperature treatments on the lifespans of the F_1_ and F_2_ generation adults. The survival rate, emergence rate, and female sex ratio were analyzed via the GLM binomial distribution. The F_2_ generation development time of the egg‐larva, cocoon, and adults did not follow a normal distribution, and the gamma distribution of the GLM was used. The F_1_ generation spawning quantity was analyzed via the GLM Poisson distribution. The survival curves of the F_1_ and F_2_ generations were drawn via GraphPad Prism 9.5 (San Diego, CA, USA), and the rest were drawn via Origin 2021 (Northampton, MA, USA).

## 3 Results

### 3.1 Effects of Different Heat Exposure Durations on the Survival Rate of Male and Female Adults

**FIGURE 3:**
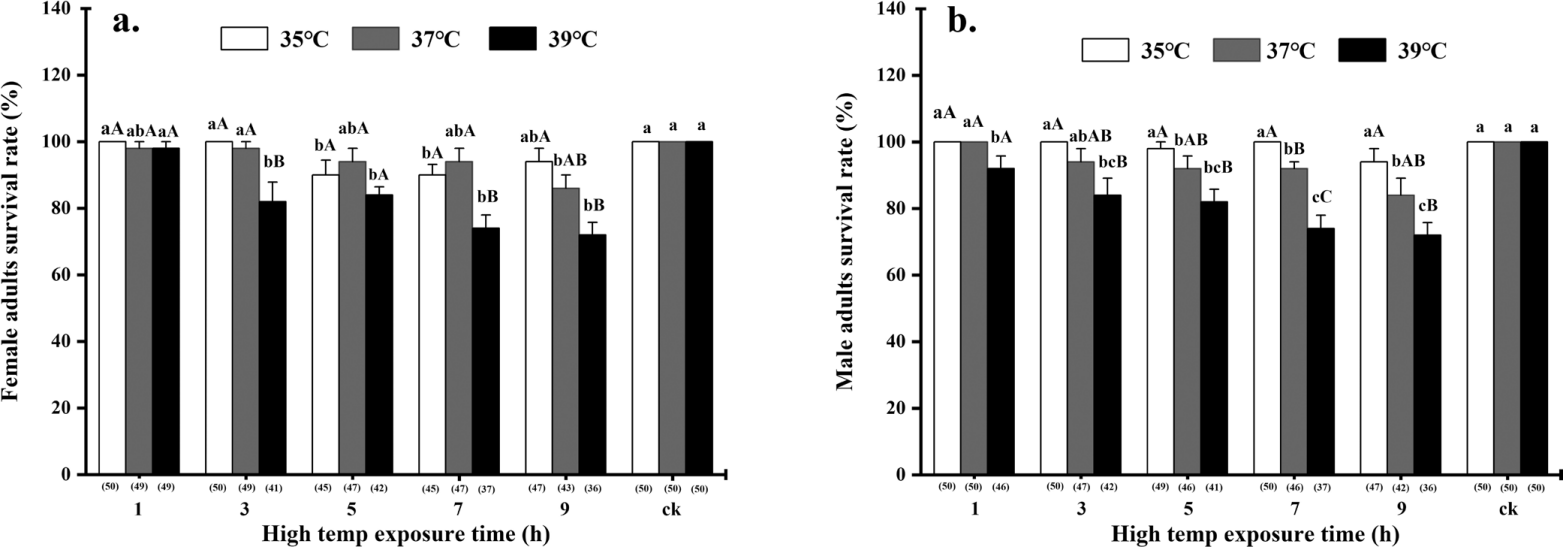
Survival rate (%) of *M. manilae* female and male adults at different high‐temperature exposure times. All values are expressed as the mean ± SEs. The numbers in parentheses below the bar chart represent the number of adults that remained alive within 1 h after different high‐temperature treatments (with an initial sample of 50 adults). The same lowercase (uppercase) letters above the means indicate no significant difference between data for different exposure times (different temperatures) at *p <* 0.05.

Under different high temperatures and exposure durations, the adult *M. manilae* exhibit a certain resistance to high temperatures, with a relatively high survival rate. Table S1 presents the effects of temperature, treatment time, sex, and their interactions on adult survival rates after exposure to high temperatures (35°C, 37°C, and 39°C) for 1, 3, 5, 7, or 9 h. Among all the factors affecting the survival rate, only temperature and treatment time had significant effects on the survival rate of female and male adults (temperature: *χ* ^2^ = 38.16, df = 2, *p* < 0.001; treatment time: *χ* ^2^ = 43.70, df = 4, *p* < 0.001), indicating that the survival of female and male adults was affected mainly by temperature and treatment time, respectively. The survival rates of female and male adults after exposure to high temperatures (35°C, 37°C, and 39°C) for 1, 3, 5, 7, or 9 h are shown in Figure 3. At the same treatment time, 39°C had the most significant effect on the survival rate of female and male adults (female: *χ* ^2^ = 31.87, df = 2, *p* < 0.001; male: *χ* ^2^ = 56.05, df = 2, *p* < 0.001). At 39°C, with increasing treatment time, the survival rates of both female and male adults decreased significantly (female: *χ* ^2^ = 58.54, df = 5, *p* < 0.001; male: *χ* ^2^ = 61.88, df = 5, *p* < 0.001). The survival rate of female and male adults treated at 39°C for 9 h was the lowest, with both being at 72.00% ± 6.35%.

### 3.2 Effects of Different High‐Temperature Exposure Times on the Lifespan of Male and Female Adults

**FIGURE 4:**
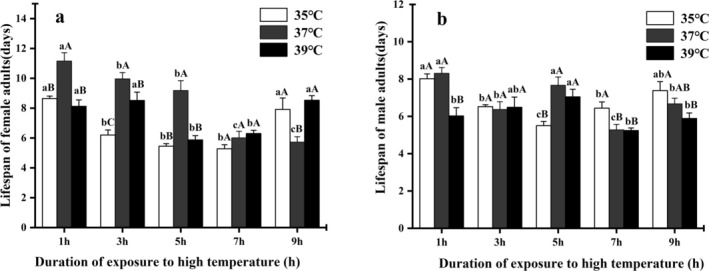
Lifespan (days) of *M. manilae* female and male adults at different high‐temperature exposure times. All values are expressed as the mean ± SEs. The same lowercase (uppercase) letters above the means indicate no significant difference between data for different exposure times (different temperatures) at *p <* 0.05. The lifespan of the control group (CK): female: 21.54 ± 1.26 (days), male: 12.68 ± 0.33 (days).

The adult lifespan of *M. manilae* was reduced after high‐temperature treatments with different exposure durations, and this reduction was more pronounced at higher temperatures. The lifespan of male and female adults still alive after exposure to high temperatures (35°C, 37°C and 39°C) for 1, 3, 5, 7, or 9 h was recorded, and analysis revealed that temperature, treatment duration, sex, and their interactions significantly affected adult lifespan (Table S2). Among the interactions among the three factors, temperature * treatment duration had the most significant effect on the lifespan of adults (*χ* ^2^ = 95.32, df = 8, *p* < 0.001). The effects of temperature and treatment duration on the lifespan of male and female adults were further analyzed (Table S3; Figure 4). At treatment durations of 1, 3, and 5 h, the survival time of female adults at 35°C and 39°C was significantly shorter than that at 37°C (1 h: *χ* ^2^ = 34.99, df = 2, *p* < 0.001; 3 h: *χ* ^2^ = 47.80, df = 2, *p* < 0.001; 3 h: *χ* ^2^ = 55.57, df = 2, *p* < 0.001). The lifespan of male and female adults after different high‐temperature treatments and different durations was much lower than that of the 26°C (CK) group. At 35°C and 39°C, with increasing treatment time, the lifespan of female adults first decreased but then increased. The lifespan of female adults treated at 35°C for 7 h was the lowest, at only 5.28 ± 0.27 days, which was significantly lower than that of the 26°C (CK) (21.54 ± 1.26 days). At different high temperatures, 39°C had the greatest effect on the lifespan of male adults, which decreased from 12.68 ± 0.33 days (CK) to 5.24 ± 0.14 days when the plants were treated at 39°C for 7 h.

### 3.3 Effect of High Temperature on the F_1_ Generation Adult Fecundity

**FIGURE 5:**
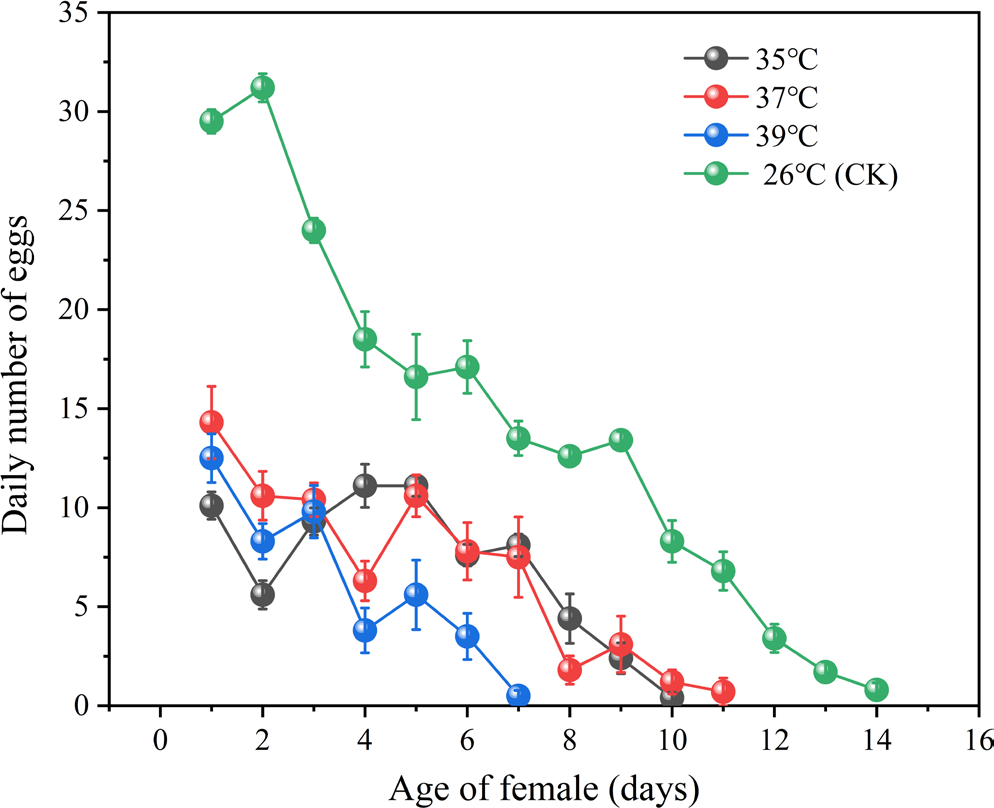
Daily number of eggs per F_1_ generation female adult of *M. manilae* under different high temperatures; all values are expressed as the mean ± SEs.

As shown in Table 1, different high temperatures predominantly had a negative effect on the fecundity of F_1_ generation adults. With increasing temperature, the number of eggs laid by female adults decreased significantly (*χ* ^2^ = 857.27, df = 3, *p* < 0.001), and the number of eggs laid at 39°C was the lowest, decreasing from 248.00 ± 4.98 eggs at 26°C (CK) to 81.90 ± 2.86 eggs. The daily egg production of female adults is shown in Figure 5. In female adults of the same age, the higher the temperature is, the lower the number of eggs. With the increasing laying age of female adults, the laying quantity of female adults at different temperatures decreased. In addition, with increasing temperature, the duration of female adult spawning gradually decreased. At 39°C, the duration of spawning decreased from 14 days at 26°C (CK) to 7 days.

**TABLE 1:** Adult fecundity (eggs) and longevity (in days) of the F_1_ generation of *M. manilae* at different high temperatures.

Temperature (°C)	Fecundity (eggs)^a^	Longevity (days)^b^				
		Female	Male	*χ* ^2^	df	*p*
35	104.60 ± 3.23c	13.60 ± 0.37bA	9.70 ± 0.56cB	29.66	1	< 0.001
37	114.10 ± 3.38b	13.40 ± 0.52bA	11.20 ± 0.61bB	9.44	1	0.002
39	81.90 ± 2.86d	9.90 ± 0.77cA	7.20 ± 0.51 dB	14.22	1	< 0.001
26 (CK)	248.00 ± 4.98a	17.50 ± 0.52aA	13.20 ± 0.25aB	36.06	1	< 0.001
*χ* ^2^	857.27	112.88	74.84			
df	3	3	3			
*p*	< 0.001	< 0.001	< 0.001			

*Note:* The data are expressed as the means ±SEs.

^a^ Means followed by the same lowercase letter in the spawning amount under different temperatures are not significantly different at *p* < 0.05.

^b^ Means followed by the same lower (upper) case letter within a column (row) are not significantly different at *p* < 0.05.

### 3.4 Effects of High Temperature on the F_1_ Generation Adult Longevity

**FIGURE 6:**
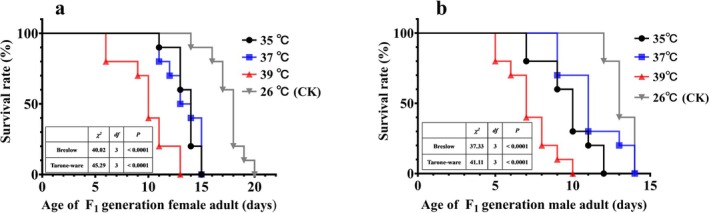
Survival curves of F_1_ generation *M. manilae* female and male adults after exposure to different high temperatures.

Table 1 shows the longevity of F_1_ generation male and female adults under different high temperatures (35°C, 37°C, and 39°C). High temperatures significantly reduced adult longevity. With increasing temperature, the longevity of both male and female adults decreased significantly (female: *χ* ^2^ = 112.88, df = 3, *p* < 0.001; male: *χ* ^2^ = 74.84, df = 3, *p* < 0.001). At 39°C, the longevity of the female adults decreased from 17.50 ± 0.52 days at 26°C (CK) to 9.90 ± 0.77 days, and the longevity of the male adults decreased from 13.20 ± 0.25 days at 26°C (CK) to 7.20 ± 0.51 days. At the same temperature, the longevity of female adults was significantly greater than that of male adults (35°C: *χ* ^2^ = 29.66, df = 1, *p* < 0.001; 37°C: *χ* ^2^ = 9.44, df = 1, *p* = 0.002; 39°C: *χ* ^2^ = 14.22, df = 1, *p* < 0.001; 26°C: (CK): *χ* ^2^ = 36.06, df = 1, *p* < 0.001). In addition, the Cox proportional hazards model was used to further evaluate the impact of different high‐temperature treatments on the lifespan of F_1_ generation adults (Table S4). The results showed that compared with the normal temperature (26°C; CK), high temperature was a risk factor for the occurrence of adult death, and the higher the temperature, the more significant the effect (*p* < 0.05). The survival curves of F_1_ generation female and male adults are shown in Figure 6. The survival rates of female and male adults decreased with increasing age of the female adults. In addition, the higher the temperature, the earlier female and male adults started to die.

### 3.5 Effects of High Temperature on the Development Time of F_2_ Generation Egg‐Larvae, Cocoons, and Adults

The development times of F_2_ generation egg‐larvae and cocoons at different temperatures are shown in Table 2. At different temperatures, the development time of egg larvae significantly increased with increasing temperature (*χ* ^2^ = 43.33, df = 3, *p* < 0.001). At 39°C, the development time increased from 7.50 ± 0.13 days at 26°C (CK) to 8.95 ± 0.18 days. The development times of cocoons at 35°C, 37°C, and 39°C were significantly shorter than that at 26°C (CK) (*χ* ^2^ = 18.68, df = 3, *p* < 0.001), but there was no significant difference among 35°C, 37°C, and 39°C. The shortest development time was at 39°C, which decreased from 5.21 ± 0.16 days at 26°C (CK) to 4.30 ± 0.15 days.

**TABLE 2:** Egg‐larva and cocoon development time (in days) and adult longevity (in days) of the F_2_ generation of *M. manilae* at different high temperatures.

Temperature (°C)	Developmental time (days)^a^		Adult longevity (days)^b^			
	Egg‐larva	Cocoon	Male	*χ* ^2^	df	*p*
35	7.96 ± 0.15b	4.57 ± 0.15b	9.98 ± 0.16bB	359.11	1	< 0.001
37	8.04 ± 0.15b	4.65 ± 0.15b	9.50 ± 0.16cB	323.38	1	< 0.001
39	8.95 ± 0.18a	4.30 ± 0.15b	6.38 ± 0.18 dB	550.43	1	< 0.001
26 (CK)	7.50 ± 0.13c	5.21 ± 0.16a	14.14 ± 0.22aB	55.08	1	< 0.001
*χ* ^2^	43.33	18.68	1067.72			
df	3	3	3			
*p*	< 0.001	< 0.001	< 0.001			

*Note:* The data are expressed as the means ±SEs.

^a^ Means followed by the same lowercase letter within a column are not significantly different at *p* < 0.05.

^b^ Means followed by the same lower (upper) case letter within a column (row) are not significantly different at *p* < 0.05.

**FIGURE 7:**
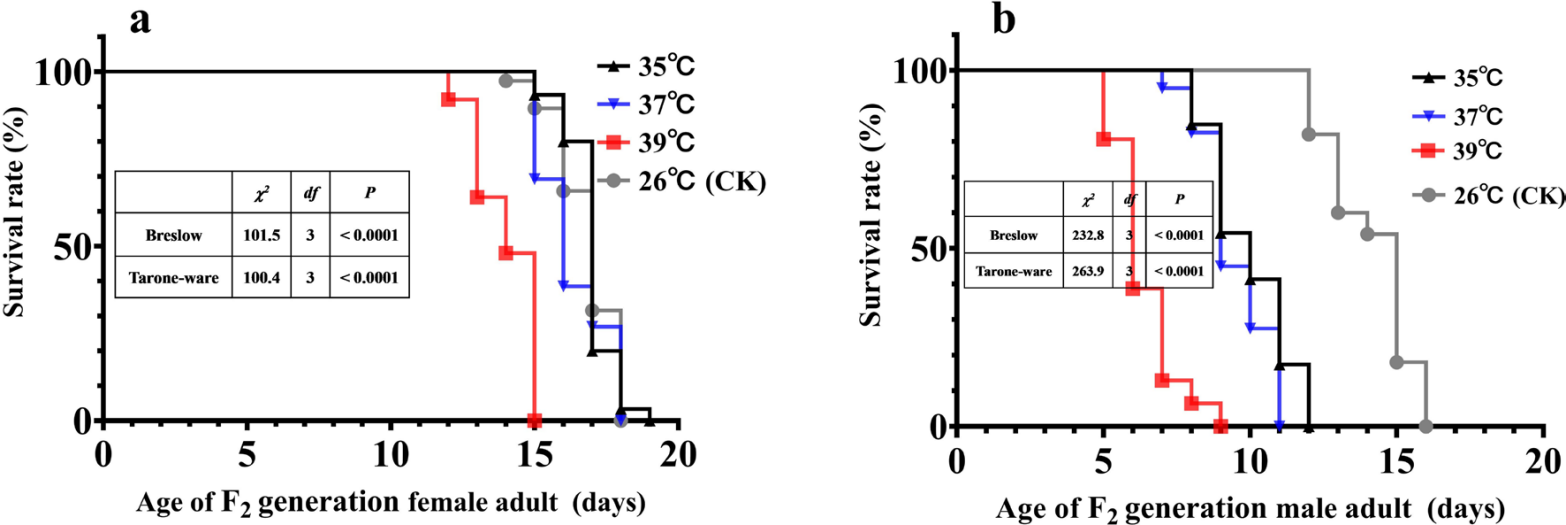
Survival curves of F_2_ generation *M. manilae* female and male adults after exposure to different high temperatures.

The effects of high temperature on the development of F_2_ generation male and female adults are shown in Table 2. The longevity of female adults at 39°C was significantly lower than that at 26°C (CK), 35°C, and 37°C (*χ* ^2^ = 1067.72, df = 3, *p* < 0.001), which was 14.04 ± 0.30 days, and there was no significant difference among those at 26°C (CK), 35°C, and 37°C. The longevity of male adults decreased gradually with increasing temperature, and the shortest longevity was 6.38 ± 0.18 days at 39°C. The longevity of female adults was significantly greater than that of male adults at different temperatures (26°C (CK): *χ* ^2^ = 55.08, df = 1, *p* < 0.001; 35°C: *χ* ^2^ = 359.11, df = 1, *p* < 0.001; 37°C: *χ* ^2^ = 323.38, df = 1, *p* < 0.001; 39°C: *χ* ^2^ = 550.43, df = 1, *p* < 0.001). In addition, the Cox proportional hazards model was used to further evaluate the impact of different high‐temperature treatments on the lifespan of F_2_ generation adults (Table S5). The results showed that compared with the normal temperature (26°C), high temperature was a risk factor for the occurrence of adult death, and the higher the temperature, the more significant the effect (*p* < 0.05). The survival curves of F_2_ generation male and female adults are shown in Figure 7. The higher the temperature, the earlier the male and female insects die. In addition, the declining trend of the survival curve of male adults was gentler than that of female adults under different high temperatures.

### 3.6 Effects of High Temperature on the F_2_ Generation Length, Width, and Weight of Cocoons

The parameters of the F_2_ generation cocoons at different high temperatures are shown in Table 3. The cocoon parameters changed significantly at different temperatures. The cocoon width, length, and weight were significantly greater at 35°C than at 37°C, 39°C, and 26°C (CK) (cocoon length: *χ* ^2^ = 195.80, df = 3, *p* < 0.001; cocoon width: *χ* ^2^ = 181.62, df = 3, *p* < 0.001; cocoon weight: *χ* ^2^ = 167.40, df = 3, *p* < 0.001). The maximum length of the cocoon was 3.92 ± 0.02 mm (35°C), and the shortest was 3.64 ± 0.02 mm (26°C; CK). The widest cocoon width was 1.58 ± 0.01 mm (35°C), and the narrowest was 1.36 ± 0.01 mm (26°C; CK). The maximum cocoon weight was 2.51 ± 0.02 mg (35°C), and the minimum weight was 2.17 ± 0.02 mg at 37°C and 26°C (CK).

**TABLE 3:** Cocoon length, width, and weight (mean ± SEs) of the F_2_ generation of *M. manilae* at different high temperatures.

Temperature (°C)	Cocoon length (mm)	Cocoon width (mm)	Cocoon weight (mg)
35	3.92 ± 0.01a	1.58 ± 0.01a	2.51 ± 0.01a
37	3.72 ± 0.01b	1.51 ± 0.01b	2.17 ± 0.03c
39	3.63 ± 0.02c	1.44 ± 0.01c	2.31 ± 0.02b
26 (CK)	3.64 ± 0.02c	1.36 ± 0.01d	2.17 ± 0.03c
*χ* ^2^	195.80	181.62	167.40
df	3	3	3
*p*	< 0.001	< 0.001	< 0.001

*Note:* Means followed by the same lowercase letter within a column are not significantly different at *p* < 0.05 (Tukey's HSD test, one‐way ANOVA).

### 3.7 Effects of High Temperature on the F_2_ Generation Adult Length of the Forewing and Hind‐Tibia

The forewing lengths of F_2_ generation adults at different high temperatures are shown in Table 4. The forewing length of both female and male adults decreased significantly with increasing temperature (female: *χ* ^2^ = 70.51, df = 3, *p* < 0.001; male: *χ* ^2^ = 75.52, df = 3, *p* < 0.001), and the forewing length of female adults decreased from 2.46 ± 0.01 mm at 26°C (CK) to 2.36 ± 0.01 mm at 39°C. The number of male adults decreased from 2.45 ± 0.01 mm at 26°C (CK) to 2.36 ± 0.01 mm at 39°C. There was no significant difference between the forewings of female and male adults at different high temperatures (26°C; CK): *χ* ^2^ = 0.89, df = 1, *p* = 0.347; 35°C: *χ* ^2^ = 3.37, df = 1, *p* = 0.066; 37°C: *χ* ^2^ = 0.14, df = 1, *p* = 0.703; 39°C; *χ* ^2^ < 0.01, df = 1, *p* = 0.961.

**TABLE 4:** Forewing length (mean ± SE) of F_2_ generation female and male adults of *M. manilae* at different high temperatures.

Temperature (°C)	Forewing length (mm)		*χ* ^2^	df	*p*
	Female	Male			
35	2.41 ± 0.01bA	2.39 ± 0.01cA	3.37	1	0.066
37	2.42 ± 0.01bA	2.42 ± 0.01bA	0.14	1	0.703
39	2.36 ± 0.01cA	2.36 ± 0.01dA	< 0.01	1	0.961
26 (CK)	2.46 ± 0.01aA	2.45 ± 0.01aA	0.89	1	0.347
*χ* ^2^	70.51	75.52			
df	3	3			
*p*	< 0.001	< 0.001			

*Note:* Means followed by the same lower (upper) case letter within a column (row) are not significantly different at *p* < 0.05.

The hind‐tibia lengths of F_2_ generation female and male adults at different high temperatures are shown in Table 5. The length of the hind‐tibia of female and male adults decreased significantly with increasing temperature (female: *χ* ^2^ = 141.90, df = 3, *p* < 0.001; male: *χ* ^2^ = 587.05, df = 3, *p* < 0.001). The maximum length of the hind‐tibia of female adults was 0.86 ± 0.01 mm at 26°C (CK) and 0.76 ± 0.01 mm at 39°C. The hind‐tibia length of male adults was the lowest at 39°C (0.65 ± 0.01 mm) and at 26°C (CK) (0.84 ± 0.01 mm). The hind‐tibia length of female adults was significantly greater than that of male adults at different temperatures (26°C; CK): *χ* ^2^ = 8.48, df = 1, *p* = 0.004; 35°C: *χ* ^2^ = 18.67, df = 1, *p* < 0.001; 37°C: *χ* ^2^ = 3.97, df = 1, *p* = 0.046; 39°C: *χ* ^2^ = 145.60, df = 1, *p* < 0.001.

**TABLE 5:** Hind‐tibia length (mean ± SEs) of F_2_ generation female and male adults of *M. manilae* at different high temperatures.

Temperature (°C)	Hind‐tibia length (mm)		*χ* ^2^	df	*p*
	Female	Male			
35	0.82 ± 0.01bA	0.79 ± 0.01bB	18.67	1	< 0.001
37	0.80 ± 0.01cA	0.78 ± 0.01bB	3.97	1	0.046
39	0.76 ± 0.01dA	0.65 ± 0.01cB	145.60	1	< 0.001
26 (CK)	0.86 ± 0.01aA	0.84 ± 0.01aB	8.48	1	0.004
*χ* ^2^	141.90	587.05			
df	3	3			
*p*	< 0.001	< 0.001			

*Note:* Means followed by the same lower (upper) case letter within a column (row) are not significantly different at *p* < 0.05.

### 3.8 Effects of High Temperature on the F_2_ Generation Egg‐Larval Survival Rate, Emergence Rate, and Female Sex Rate

The F_2_ generation egg‐larval survival rates, emergence rates, and female sex ratios at different high temperatures are shown in Table 6. With increasing temperature, both egg‐larval survival and pupal emergence rates showed slight decreases, while female sex ratios were not significantly affected. The survival rate of egg‐larvae at 37°C and 39°C was significantly lower than that at 26°C (CK) (*χ* ^2^ = 7.57, df = 3, *p* = 0.050). The lowest survival rate of egg‐larvae was 79.00% ± 4.07% at 39°C. The eclosion rate at 39°C was significantly lower than that at 26°C (CK), 35°C, and 37°C (*χ* ^2^ = 12.57, df = 3, *p* = 0.006), which was 73.00% ± 5.04%. The female sex ratio ranged from 38% ± 5.51% in the 35°C treatment to 45.00% ± 5.50% in the 26°C control treatment, but overall there were not statistically significant differences in female sex ratio across the different temperature treatments (*χ* ^2^ = 0.99, df = 3, *p* = 0.80).

**TABLE 6:** Egg‐larva survival rate, eclosion rate, and female sex rate (mean ± SE) of the F_2_ generation of *M. manilae* at different high temperatures.

Temperature (°C)	Egg‐larva survival rate (%)	Eclosion rate (%)	Female sex ratio (%)
35	86.00 ± 3.47ab	91.00 ± 3.13a	38.00 ± 5.51a
37	81.00 ± 3.92b	81.00 ± 4.32ab	39.00 ± 6.01a
39	79.00 ± 4.07b	73.00 ± 5.04b	44.00 ± 6.57a
26 (CK)	91.00 ± 2.86a	90.00 ± 3.12a	45.00 ± 5.50a
*χ* ^2^	7.57	12.57	0.99
df	3	3	3
*p*	0.050	0.006	0.80

*Note:* The data are expressed as the means ±SEs. Means followed by the same lowercase letter within a column are not significantly different at *p* < 0.05.

## 4 Discussion

Parasitic wasps play an important role in the control of pests in agricultural production (Bredlau et al. 2019). When the external environment exceeds the optimum temperature for parasitic wasps, their survival is threatened by high‐temperature stress, and the population of parasitic wasps is affected (Zhang et al. 2019). This study investigated the effects of different high temperatures and exposure times on the survival rate and the lifespan of *M. manilae* . The results revealed that 39°C had the most significant effect on the survival rate of female and male adults, and the survival rate decreased significantly with increasing exposure time. The results of this experiment are similar to those of parasitoids such as the diamondback moth pteromalid wasp *Diadegma insulare* and the diamondback moth parasitoid wasp *Cotesia vestalis*, which showed a significant decline in survival under high‐temperature stress (Bahar et al. 2013; Machekano et al. 2018). Heat kills insects directly and affects individuals who survive heat (Mironidis and Matilda 2010). The lifespan of the male and female adults in the high‐temperature treatment group was much lower than that in the 26°C (CK) control group. The results revealed that high‐temperature stress caused some damage to the insect body. When female adults were treated for less than 5 h, the lifespan at 37°C was greater than that at 35°C and 39°C. In addition, with increasing treatment time at 35°C and 39°C, the lifespan of female adults first decreased but then increased. These findings indicated that when the exposure time to high temperatures increased, *M. manilae* exhibited heat resistance. Studies have shown that when insects are stimulated by high ambient temperatures, they develop a “toxic excitatory effect”, which is an important adaptation to cope with adverse external temperatures (Villalpando et al. 2009; Hoffmann and Sgrò 2011). Therefore, this adaptive mechanism may also be present in *M. manilae* . Future research could focus on further exploring this mechanism in the species, thereby providing a theoretical framework for understanding the regulation of its environmental adaptation and evolution.

Fecundity and longevity are important indices for evaluating the reproduction and growth of insect populations (Skourti et al. 2019). When subjected to environmental stressors, the fertility of insects often declines significantly (Šupina et al. 2022). Previous research systematically evaluated the effects of exposing adults of *M. manilae* to 35°C, 37°C, and 39°C for 1–9 h on survival and lifespan. Results indicated that a 5‐h exposure at all three temperatures resulted in the most substantial decline in lifespan, with post‐heat‐treatment survival rates exceeding 80% (Figure 3). Building on these findings, this study maintained a 5‐h exposure duration across the same temperature gradients to investigate the influence of heat stress on F_1_‐generation fecundity and post‐reproductive lifespan of *M. manilae* . The results revealed that with increasing temperature, the fecundity of female adults and the longevity of male and female adults decreased significantly. These results are similar to those of the Achaean t*richogramma* wasp *Trichogramma achaeae* and the pine caterpillar *trichogramma* wasp *Trichogramma dendrolimi*, which presented significant decreases in fecundity and longevity at high temperatures (Zhou et al. 2019; Modesto et al. 2020). These results indicated that short‐term high temperatures are not favorable for the fecundity and longevity of adult *M. manilae* . Therefore, the effects of external environmental weather extremes on insects during the field release of natural enemies should be given more importance, perhaps by avoiding the release in high‐temperature weather to reduce the effect of high temperatures on the field biological control of natural enemies (Zhen et al. 2021; Jackson et al. 2022).

Short‐term heat treatment of insect parents usually affects the growth and development of offspring. This phenomenon is called the “maternal effect”, which is a type of nongenetic effect of the mother on the offspring (Mousseau and Fox 1998). In this study, to investigate the transgenerational effects of high‐temperature stress, the F_2_ generation larvae were subjected to the same temperature treatments as those for the parental generation. We monitored the F_2_ generation egg‐larval stages after different high‐temperature treatments of F_1_ adults and found a significant prolongation of the F_2_ generation egg‐larval stages when the high temperature reached 39°C. Additionally, the cocoon stage and the adult stage were significantly shortened, which indicated that extremely high temperatures hindered the development of the F_2_ generation of *M. manilae* (Pan et al. 2024).

The effect of high temperatures on the F_2_ generation cocoon parameters was greatest at 35°C, with an increase in all the parameters relative to those of the control group, but the parameters initially decreased as the temperature increased. This may be related to the self‐regulatory mechanisms that occur in insects as the ambient temperature changes (Daniel et al. 2020). In addition, after the adults of the F_1_ generation were subjected to different high temperatures, the survival rate of the egg larvae and the emergence rate of the cocoon of the F_2_ generation significantly decreased at 39°C. These results are similar to those reported for most parasites under heat stress (Malinski et al. 2024). However, the female sex ratio did not change significantly with heat, and the results indicated that the F_2_ generation of *M. manilae* was somewhat stable to high temperatures in terms of the sex ratio of females; a similar phenomenon was also observed in *D. melanogaster* (Min et al. 2021). The development of forewing and hind‐tibia lengths in adult insects can reflect the development of adult individuals to some extent (Stephens and Juliano 2012).

In this study, after high‐temperature stress in F_1_ generation adults, both the forewing and hind‐tibial lengths of F_2_ generation male and female adults decreased as the temperature increased, indicating that the ontogeny of F_2_ generation adults was also inhibited by high temperature. These results are remarkably similar to those shown by parasitoids such as the grain aphid parasitoid wasp *Aphidius rhopalosiphi* and the white‐winged *pteromalus* wasp *Pteromalus albipennis* at high temperatures (Cécilele et al. 2011; Xi et al. 2017). These results showed that short‐term high temperatures affected the development of the F_1_ and F_2_ generations, further highlighting the important role of environmental factors in the regulation of the growth and development of insects (Verma et al. 2024).

To summarize, in this study, we elucidated the effects of short‐term high temperatures on the biological characteristics of different generations of *M. manilae* at the biological level. Building on the research by Qiu et al. (2012), which explored the effects of suitable temperatures ranging from 17°C to 32°C on the biological characteristics of *M. manila*, our study further fills the gap in the research field regarding the effects of high‐temperature environments on the biological characteristics of *M. manila*. However, this study merely investigated the effects of high‐temperature stress on the macroscopic biological traits of *M. manilae* . The molecular regulatory mechanisms underlying its thermotolerance under high‐temperature stress remain unexplored. Therefore, future research should focus on elucidating how short‐term high‐temperature stress impacts the molecular biological properties of *M. manilae* . Such investigations will be instrumental in providing a scientific foundation for employing *M. manilae* in pest biocontrol during high‐temperature seasons.

## Author Contributions

**Peng Ren:** data curation (equal), methodology (equal), writing – original draft (equal), writing – review and editing (equal). **Xue‐Yuan Di:** conceptualization (equal), methodology (equal). **Bin Yan:** funding acquisition (equal), supervision (equal). **Xiu‐Xian Shen:** conceptualization (equal), methodology (equal). **Shuang Tian:** data curation (equal), formal analysis (equal). **Mao‐Fa Yang:** funding acquisition (equal), supervision (equal), writing – review and editing (equal). **Hui‐Zi Wu:** funding acquisition (equal), methodology (equal), supervision (equal).

## Conflicts of Interest

The authors declare no conflicts of interest.

## Supporting information


Appendix S1.

## Data Availability

The research data of this study are stored at Dryad. Journal editors and peer reviewers are permitted to access the data for the purpose of reviewing and evaluating this manuscript submission. The data are intended solely for this academic purpose. Moreover, the data storage is secure, with secure data encryption techniques employed and relevant laws and regulations followed to ensure data integrity and privacy. Data reference: “Raw data” “Supporting Information”, Dryad, doi: https://doi.org/10.5061/dryad.b5mkkwhq9.
