# Supplementary material for: Impact of Short‐Term High Temperature on Microplitis manilae (Hymenoptera: Braconidae) Survival, Fecundity and Progeny Development
Source: Ecol Evol. 2025 Jun 18;15(6):e71626. doi: 10.1002/ece3.71626 (PMC12176452; doi:10.1002/ece3.71626)
Supplement: Supplementary file 1 — Appendix S1. [file ECE3-15-e71626-s001.docx]

**Table S1.** Model effect test for the influence of temperature, treatment time, sex, and their interaction on the survival rate of *M. manilae* adults.

| Source | *df* | *χ^2^* | *P* value |
| --- | --- | --- | --- |
| Temperature | 2 | 38.16 | < 0.001 |
| Sex | 1 | < 0.01 | 0.992 |
| Treatment time | 4 | 43.70 | < 0.001 |
| Temperature * Sex | 2 | 0.61 | 0.736 |
| Temperature * Treatment time | 8 | 10.22 | 0.250 |
| Sex * Treatment time | 4 | 3.03 | 0.553 |
| Temperature * Sex * Treatment time | 8 | 8.47 | 0.389 |

Results table of the likelihood ratio chi-square test for the influence of different factors on survival rate.

**Table S2.** Model effect test for the influence of temperature, treatment time, sex, and their interaction on the survival time of *M. manilae* adults.

| Source | *df* | *χ^2^* | *P* value |
| --- | --- | --- | --- |
| Temperature | 2 | 35.14 | < 0.001 |
| Sex | 1 | 44.77 | < 0.001 |
| Treatment time | 4 | 114.41 | < 0.001 |
| Temperature * Sex | 2 | 28.26 | < 0.001 |
| Temperature * Treatment time | 8 | 95.32 | < 0.001 |
| Sex * Treatment time | 4 | 30.36 | < 0.001 |
| Temperature * Sex * Treatment time | 8 | 51.98 | < 0.001 |

Results table of the likelihood ratio chi-square test for the influence of different factors on survival time.

**Table S3.** Lifespan (in days) of *M. manilae* adults at different high-temperature exposure times.

| Sex | Treatment time (h) | Temperature (°C) | | | *χ^2^* | *df* | *P* value |
| --- | --- | --- | --- | --- | --- | --- | --- |
|  |  | 35 | 37 | 39 |  |  |  |
| Female | 1 | 8.64 ±0.17aB | 11.15 ±0.56aA | 8.12 ±0.43aB | 34.99 | 2 | < 0.001 |
|  | 3 | 6.20 ±0.34bC | 9.96 ±0.43bA | 8.51 ±0.56aB | 47.80 | 2 | < 0.001 |
|  | 5 | 5.45 ±0.18bB | 9.18 ±0.67bA | 5.87 ±0.28bB | 55.57 | 2 | < 0.001 |
|  | 7 | 5.28 ±0.27bA | 6.01 ±0.44cA | 6.30 ±0.21bA | 3.70 | 2 | 0.157 |
|  | 9 | 7.91 ±0.76aA | 5.72 ±0.37cB | 8.53 ±0.31aA | 28.99 | 2 | < 0.001 |
|  | *df* | 4 | 4 | 4 |  |  |  |
|  | *χ^2^* | 60.42 | 156.57 | 43.53 |  |  |  |
|  | *P* | < 0.001 | < 0.001 | < 0.001 |  |  |  |
| Male | 1 | 8.02 ±0.25aA | 8.30 ±0.31aA | 6.02 ±0.44bB | 30.78 | 2 | < 0.001 |
|  | 3 | 6.52 ±0.10bA | 6.37 ±0.42bA | 6.48 ±0.55abA | 0.13 | 2 | 0.937 |
|  | 5 | 5.50 ±0.22cB | 7.67 ±0.44aA | 7.04 ±0.41aA | 24.67 | 2 | < 0.001 |
|  | 7 | 6.44 ±0.33bA | 5.27 ±0.29cB | 5.24 ±0.14bB | 9.29 | 2 | 0.010 |
|  | 9 | 7.39 ±0.48abA | 6.67 ±0.30bAB | 5.89 ±0.30bB | 11.23 | 2 | 0.004 |
|  | *χ^2^* | 37.01 | 55.02 | 18.13 |  |  |  |
|  | *df* | 4 | 4 | 4 |  |  |  |
|  | *P* | < 0.001 | < 0.001 | 0.001 |  |  |  |

The data are expressed as the means ±SEs (days). Means followed by the same lower (upper) case letter within a column (row) are not significantly different at *P* < 0.05. The lifespan of the control group (CK): Female: 21.54 ±1.26 (days), Male: 12.68 ±0.33 (days).

**Table S4.** Cox Proportional Hazards analysis of the effect of temperature on the lifespan of male and female adult F_1_ generation *M. manilae*

| Temperature | Regression coefficient (β) | Standard error (SE) | Wald χ^2^ | *df* | *P* | HR | HR (95% CI) | |
| --- | --- | --- | --- | --- | --- | --- | --- | --- |
|  |  |  |  |  |  |  | Lower limit | Upper limit |
| 26℃ |  |  | 57.079 | 3 | < 0.001 |  |  |  |
| 35℃ | 1.860 | 0.400 | 21.566 | 1 | < 0.001 | 6.421 | 2.929 | 14.076 |
| 37℃ | 1.220 | 0.377 | 10.440 | 1 | 0.001 | 3.386 | 1.616 | 7.095 |
| 39℃ | 3.412 | 0.458 | 55.435 | 1 | < 0.001 | 30.328 | 12.353 | 74.461 |

**Table S5.** Cox Proportional Hazards models analysis of the effect of temperature on the lifespan of male and female adult F_2_ generation *M. manilae*

| Temperature | Regression coefficient (β) | Standard error (SE) | Wald χ^2^ | *df* | *P* | HR | HR (95% CI) | |
| --- | --- | --- | --- | --- | --- | --- | --- | --- |
|  |  |  |  |  |  |  | Lower limit | Upper limit |
| 26℃ |  |  | 185.251 | 3 | < 0.001 |  |  |  |
| 35℃ | 1.376 | 0.201 | 46.919 | 1 | < 0.001 | 3.959 | 2.670 | 5.868 |
| 37℃ | 1.656 | 0.212 | 61.145 | 1 | < 0.001 | 5.238 | 3.458 | 7.932 |
| 39℃ | 3.547 | 0.262 | 183.213 | 1 | < 0.001 | 34.710 | 20.768 | 58.011 |
